# Editorial: New Insights Into the Complexity of Tumor Immunology in B-Cell Malignancies: Prognostic and Predictive Biomarkers and Therapy

**DOI:** 10.3389/fonc.2021.841763

**Published:** 2022-01-17

**Authors:** Etienne Moussay, Martina Seiffert, Jérôme Paggetti

**Affiliations:** ^1^ Tumor-Stroma Interactions, Department of Cancer Research, Luxembourg Institute of Health, Luxembourg, Luxembourg; ^2^ Molecular Genetics, German Cancer Research Center (DKFZ), Heidelberg, Germany

**Keywords:** leukemia, tumor microenvironment, therapy, anti-tumor immunity, biomarkers, lymphoma

The importance of the tumor microenvironment (TME) in sustaining tumor growth is nowadays well described in hematologic malignancies. The aim of the Research Topic is to summarize for the reader some recent advances related to biomarkers and therapies in B-cell leukemias and lymphomas including multiple cell types, soluble factors, and extracellular vesicles (EV) found in the TME. Specifically, Ondrisova and Mraz describe the role of the TME in triggering pro-proliferative and anti-apoptotic signals for chronic lymphocytic leukemia (CLL) cells and the respective contributions of T cells, stromal cells, and monocytes/nurse-like cells in the secretion of soluble factors. They review the current therapeutic interventions with a focus on ibrutinib and idelalisib, and discuss the associated resistance mechanisms and strategies to overcome them.

Advances for the development of new targeted therapies focused in recent years on B-cell receptor signaling and induction of apoptosis. In addition to ibrutinib and idelalisib, venetoclax (Bcl-2 inhibitor) completes the arsenal of targeted therapies for B-cell malignancies. Bcl-2 inhibition is currently used as first-line therapy and for the treatment of relapsed/refractory CLL and acute myeloid leukemia patients. In this topic issue, Vereertbrugghen et al. show the sensitivity of hairy cell leukemia cells to venetoclax *in vitro*, independently of the disease form (classic, variant, and VH4-34). However, the protection of stromal cells observed in co-culture points to the need to target both leukemic cells and the microenvironment.

Several solid cancers, including from pancreas, breast, and lung, show important signs of inflammation. Since many years, a high systemic immune-inflammation index (SII), associated with neutrophil, platelet, and lymphocyte counts, is reported as a poor prognostic marker. Wang et al. perform a retrospective study on 224 patients to evaluate the power of SII in diffuse large B-cell lymphoma (DLBCL). They compare SII with the pretreatment neutrophil-lymphocyte ratio (NLR) and the platelet-lymphocyte ratio (PLR) reported to be associated with disease outcome. In multivariate analyses, a high SII correlates with a poor overall survival, demonstrating its suitability as an accurate prognostic factor.

The TME is a very plastic entity, being under the influence of the tumor and therefore being constantly remodeled. In this topic, Menzel et al. extensively describe the angiogenic process as a major component of TME remodeling in lymph nodes (LN). The authors describe the vasculature as a major contributor to inflammation and cancer development in multiple lymphoma subtypes. Very interestingly, although crucial for anti-tumor immune response, immune cells also contribute to the production of neoangiogenic factors. The endothelium being crucial for effective immune infiltration, the authors discuss the careful design of therapeutic strategies to limit neoangiogenesis without hindering the success of emerging cellular immunotherapies. The myeloid compartment has also recently gained interest in lymphoma. Ferrant et al. precisely describe the diversity of myeloid derived suppressor cells of monocytic and polymorphonuclear origins, their phenotypes and their functional roles and identify S100A9^high^ circulating myeloid cells as promising diagnostic and prognostic biomarkers in DLBCL.

Tumor-derived EV, also called exosomes, contribute as well to the plasticity of the TME, by transferring RNA and protein to target cells and by affecting cellular signaling through direct ligand-receptor interactions. Due to their characteristics and content, EV can be used as potential biomarkers to allow an early detection of tumor cells in an organism. Gargiulo et al. review the advances in the field of EV diagnostics, how EV contribute to immune evasion, and finally discuss recent strategies using engineered EV as a valuable therapeutic tool against cancer and specifically B-cell malignancies.

The easy detection of surface receptors makes them highly appreciated for biomarker development. The transmembrane glycoprotein CD200 is overexpressed by CLL cells and plays an important role in disease development and progression. D’Arena et al. describe CD200 structure, physiologic functions and its immunosuppressive role towards T cells in cancer. Based on an extensive review of the literature on CD200 expression in patients with B-cell malignancies, the authors discuss the relevance of CD200 for the diagnosis of CLL and how it helps to discriminate CLL from similar entities (e.g. mantle cell lymphoma), and its potential prognostic role.

The recurrent mutations in the splicing factor SF3B1 are associated with a defect in DNA damage response (DDR) and the generation of cryptic transcripts in CLL which are subject to degradation *via* nonsense-mediated mRNA decay (NMD). Using a cohort of treatment naïve CLL patients, Leeksma et al. study the DDR response to irradiation in SF3B1 mutated CLL patients and highlight the influence of prior treatments in their previous findings based on a mixed treatment cohort. In addition, they detect cryptic transcripts in CLL cells and activation of NMD, and therefore suggest that NMD modulatory agents can benefit patients with mutant SF3B1. Understanding the interactions between cancer cells and the TME is crucial for efficient targeting. In an effort to identify ligand-receptor pairs associated with B-cell precursor acute lymphoblastic leukemia, Wu et al. analyze RNA-sequencing data for survival analysis and prognostic model construction. The authors identify 57 ligand-receptor pairs in the autocrine network of B cells and 29 other pairs related to the communication with myeloid cells, some of which could be linked with survival outcomes. The analysis of large cohorts is valuable for the design of future therapeutic interventions. The *IGHV* mutational status has a high prognostic value and contributes to the determination of optimal treatments in CLL patients. In this issue, Bagnara et al. perform deep DNA-sequencing in CLL leukemic clones and confirm the high intraclonal *IGHV-IGHD-IGHJ* diversification. But surprisingly, the presence of subclones was similar among *IGHV* mutated and unmutated CLL patients. The expansion of subclones appears downstream of the dominant clone. The authors then intensely discuss the mechanisms involved in targeting the immunoglobulin loci and the different microenvironmental inputs in the CLL LN leading to dysregulated B-cell functions and aggressive disease.

This Research Topic brings together critical reviews and original research articles describing the TME in B-cell malignancies, reporting on the use of new genetic and non-genetic biomarkers having prognostic and diagnostic values, and suggesting their use to optimize the response to therapies for patients.

## Author Contributions

All authors edited the Research Topic. EM wrote the editorial. All authors contributed to the article and approved the submitted version.

## Funding

This work was supported by grants from the Fonds National de la Recherche Luxembourg to JP and EM (C20/BM/14592342 and C20/BM/14582635).

## Conflict of Interest

The authors declare that the research was conducted in the absence of any commercial or financial relationships that could be construed as a potential conflict of interest.

## Publisher’s Note

All claims expressed in this article are solely those of the authors and do not necessarily represent those of their affiliated organizations, or those of the publisher, the editors and the reviewers. Any product that may be evaluated in this article, or claim that may be made by its manufacturer, is not guaranteed or endorsed by the publisher.

